# Transdifferentiation of Human Circulating Monocytes Into Neuronal-Like Cells in 20 Days and Without Reprograming

**DOI:** 10.3389/fnmol.2018.00323

**Published:** 2018-09-19

**Authors:** Alfredo Bellon, Amelie Wegener, Adam R. Lescallette, Michael Valente, Seung-Kwon Yang, Robert Gardette, Julien Matricon, Faycal Mouaffak, Paula Watts, Lene Vimeux, Jong K. Yun, Yuka Imamura Kawasawa, Gary A. Clawson, Elisabeta Blandin, Boris Chaumette, Therese M. Jay, Marie-Odile Krebs, Vincent Feuillet, Anne Hosmalin

**Affiliations:** ^1^Penn State Hershey Medical Center, Department of Psychiatry, Hershey, PA, United States; ^2^Penn State Hershey Medical Center, Department of Pharmacology, Hershey, PA, United States; ^3^INSERM U1016, Institut Cochin, Paris, France; ^4^CNRS UMR8104, Paris, France; ^5^Université Paris Descartes, Sorbonne Paris Cite, Paris, France; ^6^INSERM UMR894, Center for Psychiatry and Neurosciences, Paris, France; ^7^Centre Hospitalier Sainte-Anne, Faculté de Médecine Paris Descartes, Service Hospitalo-Universitaire-S14, Paris, France; ^8^Sky Ridge Medical Center, Department of Internal Medicine, Lone Tree, CO, United States; ^9^Penn State Hershey Medical Center, Department of Biochemistry and Molecular Biology, Institute for Personalized Medicine, Hershey, PA, United States; ^10^Gittlen Cancer Research Laboratories, Department of Pathology, Penn State University College of Medicine, Hershey, PA, United States; ^11^Penn State Hershey Medical Center, Neural & Behavioral Sciences, Hershey, PA, United States; ^12^Montreal Neurological Institute and Hospital, Department of Neurology and Neurosurgery, McGill University, Montreal, QC, Canada

**Keywords:** stem cells, dopamine, schizophrenia, neurite, *in vitro* model, GABA, neurodevelopment, autism

## Abstract

Despite progress, our understanding of psychiatric and neurological illnesses remains poor, at least in part due to the inability to access neurons directly from patients. Currently, there are *in vitro* models available but significant work remains, including the search for a less invasive, inexpensive and rapid method to obtain neuronal-like cells with the capacity to deliver reproducible results. Here, we present a new protocol to transdifferentiate human circulating monocytes into neuronal-like cells in 20 days and without the need for viral insertion or reprograming. We have thoroughly characterized these monocyte-derived-neuronal-like cells (MDNCs) through various approaches including immunofluorescence (IF), flow cytometry, qRT-PCR, single cell mRNA sequencing, electrophysiology and pharmacological techniques. These MDNCs resembled human neurons early in development, expressed a variety of neuroprogenitor and neuronal genes as well as several neuroprogenitor and neuronal proteins and also presented electrical activity. In addition, when these neuronal-like cells were exposed to either dopamine or colchicine, they responded similarly to neurons by retracting their neuronal arborizations. More importantly, MDNCs exhibited reproducible differentiation rates, arborizations and expression of dopamine 1 receptors (DR1) on separate sequential samples from the same individual. Differentiation efficiency measured by cell morphology was on average 11.9 ± 1.4% (mean, SEM, *n* = 38,819 cells from 15 donors). To provide context and help researchers decide which *in vitro* model of neuronal development is best suited to address their scientific question,we compared our results with those of other *in vitro* models currently available and exposed advantages and disadvantages of each paradigm.

## Introduction

The inability to access neurons directly from patients is a major obstacle to understanding psychiatric and neurological illnesses at a cellular level. This limitation is currently being circumvented by employing either various types of stem cells or samples from the olfactory neuroepithelium. Each of these approaches carries its own set of advantages and disadvantages.

There are technical but above all, ethical concerns surrounding the retrieval and utilization of human embryonic stem cells (ESC; de Wert and Mummery, 2003). For many researchers and legislators, obtaining human embryos for the sole purpose of isolating stem cells poses a moral question (Young, 2000). Not surprisingly, these controversies have prompted research into alternative approaches, one of which produced the unexpected possibility of generating pluripotent stem cells from already differentiated adult cells (Takahashi and Yamanaka, 2006). The advent of induced pluripotent stem cells (IPSCs) has generated tremendous enthusiasm in the scientific community and these cells are already a widely used research tool. But IPSCs (just as any other model) have limitations. Reprograming adult somatic cells into IPSCs involves altering the cell’s genome via viral insertion (Takahashi and Yamanaka, 2006), although other non-integrative techniques are currently in use such as, episomal vectors and the non-integrative sendai virus. This process can elicit genetic and epigenetic abnormalities (Urbach et al., 2010; Pera, 2011) which could become confounders when trying to understand psychiatric and neurological illnesses at a cellular level (Bellin et al., 2012). These confounders could become even more significant when studying alignments with strong, but still poorly understood, genetic components such as schizophrenia and autism. Another significant concern with IPSCs is the lack of reproducible results with samples from the same individual (Hu et al., 2010; Dolmetsch and Geschwind, 2011). This is particularly important when IPSCs are used to compare cells from patients vs. controls as variability in cell lines from the same person hinders our ability to draw firm conclusions about disease phenotypes (Bellin et al., 2012). In addition, generating IPSCs is costly and time-consuming (Dolmetsch and Geschwind, 2011; Borgmann-Winter et al., 2015; Petersen and Strappe, 2016) and, as a result, only very few patients can be studied simultaneously. The stem cell field is starting to realize that IPSCs are an extraordinary model for monogenetic illnesses where clear mutations can be identified and their phenotype pursued. But this type of stem cell is significantly less helpful for studying complex multigenetic illnesses with undetermined genetic backgrounds such as most psychiatric and neurological disorders.

Adult stem cells and olfactory neuroepithelial cells (ONCs) have also received significant and well-deserved attention as *in vitro* models. Among the most commonly utilized adult stem cells are mesenchymal stem cells (MSCs). MSCs are considered non-immunogenic (Chamberlain et al., 2007). This characteristic has driven research with these cells mostly toward exploring their treatment potential, in particular, as cellular transplants (Taran et al., 2014). Their use to study psychiatric and neurological disorders is very limited, even though MSCs can be readily differentiated into neuronal lineages without the need for reprograming (Woodbury et al., 2000). MSCs can be obtained from various sources ranging from adipose tissue to dental pulp (Taran et al., 2014). But in the neuroscientific field the most common origin is bone marrow (Taran et al., 2014). Their limited use to model brain disorders *in vitro* seems to be related to accessibility, as a bone marrow sampling is an invasive and painful procedure. Hence, bone marrow-MSCs (BM-MSCs) research has been mostly directed to regenerative medicine (Taran et al., 2014).

Another available model to study brain illnesses *in vitro* are ONCs. ONCs can be rapidly and inexpensively obtained and do not require viral insertion into the cells’ genome (Borgmann-Winter et al., 2015). In addition, this is the only approach currently available that can provide actual neurons (Borgmann-Winter et al., 2009). Some authors even suggest ONCs could offer information about limbic regions because of the anatomical areas involved within the olfactory circuit (Borgmann-Winter et al., 2015). Similar to BM-MSCs, ONCs limitation relates to availability, as access to the neuroepithelium requires either a biopsy of the olfactory mucosa (Féron et al., 1998) or a special brush that reaches deep into the nasal cavity (Benítez-King et al., 2011). Both of these techniques are invasive procedures that should be performed by a qualified otorhinolaryngologist. Concerns have also been raised about the heterogeneity of cells obtained with each biopsy as they range from epithelial to glial to neuroprogenitor cells as well as neurons at different stages of differentiation (Féron et al., 1998; Borgmann-Winter et al., 2015). This diversity of cell types could become a confounder when comparing cells from patients vs. controls since biopsies from even the same individual can produce variable results (Féron et al., 1998; Borgmann-Winter et al., 2015). Therefore, the search for more practical and less invasive methods to obtain neuronal-like cells that can deliver reproducible results continues. A nascent alternative is transdifferentiation of circulating monocytes.

In 2003, two independent teams demonstrated that a subset of human circulating monocytes had pluripotent capacities (Kuwana et al., 2003; Zhao et al., 2003). Since then, several groups have shown that human monocytes can express neuronal markers if cultured under appropriate conditions (Zhao et al., 2003; Romagnani et al., 2005; Porat et al., 2006). But only two teams have been able to produce cells that structurally resemble neurons (Kodama et al., 2006; Horschitz et al., 2010) and one of these protocols required co-culturing human monocytes with rat neurons (Kodama et al., 2006). Expression of neuronal markers and structural resemblance to neurons is a remarkable first step but to date, comprehensive evidence that human monocytes can be transdifferentiated into the neuronal lineage is still lacking. Moreover, what the scientific community requires is a protocol that can be easily and consistently reproduced. Here, we present extensive evidence from a variety of approaches that supports the potential for a subset of human circulating monocytes to differentiate into neuronal-like cells. In addition, we have tested our transdifferentiation protocol in 68 individuals in two different independent laboratories located in separate continents (Table 1) and obtained comparable results. Finally, we compared and contrasted this new model with the other *in vitro* models currently available.

**Table 1:** Total number of donors providing blood samples transdiffrentiated into neuronal-like cells.

Experiment	Figure	Location of laboratory	Donors* providing blood samples	Age range	Gender
Cell viability	1B	Paris, France	10	20 to 59	7 males 3 females
Characterization of structural stages before transdifferentiation	1C	Paris, France	15	19 to 67	12 males 3 females
Expression of CD14 and CD34 by flow cytometry	1E,F	Paris, France	12	20 to 55	9 males 3 females
BrdU incorporation	1E	Hershey, Pennsylvania, USA	6	26 to 42	3 males 3 females
Expression of CD14 by immunofluorescence	1F	Paris, France	3	40 to 55	2 males 1 female
Immunofluorescence with actin and tubulin	2C	Paris, France	5	25 to 27	4 males 1 female
Expression of neuronal markers by immunofluorescence	2D–H	Paris, France	7	22 to 49	5 males 2 females
Expression of neuronal, glial and stem cell markers by flow cytometry	2I	Paris, France	8	23 to 61	7 males 1 female
Expression of neuronal and glial genes by qRT-PCR	3A	Paris, France	6	20 to 60	5 males 1 female
Single cell mRNA sequencing	3B, Supplementary Table S1**	Hershey, Pennsylvania, USA	1	24	1 female
Electrophysiology by patch clamp	4A–F	Paris, France	5	22 to 30	4 males 1 female
Structural responses to colchicine and dopamine	5A–D	Paris, France	4	26 to 42	3 males 1 female
Reproducible results with MDNCs***	6A–G	Hershey, Pennsylvania, USA	4	26 to 42	4 males
Differentiation efficiency by cell morphology from EDTA tubes	Not in figures	Paris, France	15	19 to 67	12 males 3 females
Differentiation efficiency by cell morphology from leucoreduction filters	Not in figures	Paris, France	3	40 to 51	2 males 1 female
Expression of GAD****by qRT-PCR	Supplementary Figure S1	Hershey, Pennsylvania, USA	3	25 to 44	1 male 2 females
Characterization of late structural stages leading to neuronal-like cells	Supplementary Figure S2	Paris, France	2	64 to 67	2 males
Total number of donors which blood samples were transdiffrentiated into neuronal-like cells = 68					

*Some of the cells obtained from a single donor were used for more than one type of experiment, **Supplementary, ***monocyte-derived-neuronal-like cells, ****Glutamic acid decarboxylase.

## Materials and Methods

### Cell Culture

Fresh blood was obtained from healthy individuals. All participants, after receiving a full description of the study, gave their informed and written consent. All study procedures were approved by local ethics committees (INSERM and Penn State University STUDY00006911) and were in accordance with the Helsinki Declaration. Three different blood collection methods were tested; EDTA (purple) tubes, leucoreduction filters (in France only, in the USA leucoreduction filters hinder cell extraction) and whole blood bags. Blood collected in leucoreduction filters and whole blood bags were obtained from the Blood Bank at the Saint-Vincent de Paul Hospital, Paris, France following an ethics convention with INSERM. Blood samples were processed within 24 h and when possible shortly after extraction. A total of 68 donors were recruited for this study (46 males and 22 females) the age range was 19–67. All donors recruited to this study are listed in Table 1.

Blood components were separated by Ficoll-Paque (GE Healthcare, 17-1440-03). A fraction of peripheral blood mononuclear cells (PBMCs) were washed and cultured on fibronectin-coated 25 cm^2^ flasks. We cultured 13.5 million PBMCs per 25 cm^2^ flask. The remaining PBMCs were used for isolation of CD14^+^ cells (monocytes) by positive immunomagnetic selection (CD14 human Microbeads, Miltenyi Biotec, 130-050-201). CD14^+^ cells were cultured on fibronectin-coated wells or flasks at a concentration of 180,000 cells per cm^2^. Plastic plates and flasks came from BD Falcon (351146, 353043 and 353109). Human fibronectin from plasma (Sigma-Aldrich, F2006) was used at a concentration of 20 μg/ml and coating was done overnight at 4°C. Macrophage colony-stimulating factor (MCSF) from AbCys (300-25) was added to monocytes right before culturing. MCSF was used at a final concentration of 50 ng/ml. All cells were maintained in Dulbecco’s Modified Eagle Medium (DMEM), High Glucose, GlutaMAX (GIBCO, 61965059) in which we added 100 U/mL penicillin; 100 mg/mL streptomycin, 1% nonessential amino acids, 1 mM sodium pyruvate, 10 mM HEPES buffer (all from Life Technologies) and supplemented with 10% fetal bovine serum (FBS) from GIBCO Performance Plus (in Europe 10270-106 and in the USA 16000, Life Technologies). In the experiments made in the USA, two different batches of this serum gave reproducible results, while others not. In the experiments made in France a panel of five other FBS from different providers was tested. As often is the case for cell culture, FBS screening is recommended before starting a batch of cultures, at least, on the basis of yield, viability and neuronal-like morphology.

No decomplementation protocols were followed to treat FBS and we do not recommend following any such protocols. For comparisons of two blood samples from the same individual at two different time points we used the same lot of FBS. After 4 days in culture at 37°C with 5% CO_2_ pressure, cell media was changed. Supplemented-DMEM as well as PBMCs-conditioned media at a rate of 2:1 (supplemented-DMEM to PBMCs-conditioned media) were used to replace old media. PBMCs-conditioned media was obtained by recovering media from PBMCs cultured in parallel from the same individual. PBMCs-conditioned media was centrifuged 1,200 rpm for 7 min at room temperature and then heated at 37°C before use. On day 7, cultured media was replaced again but now at a ratio of 1:1 (PBMCs-conditioned media to supplemented-DMEM). Butylated hydroxyanisole (BHA; Sigma-Aldrich, B1253) was added to a final concentration of 50 nM. Replacement of cell culture media on day 10 was similar to day 7. The ratio PBMCs-conditioned media to DMEM is also 1:1 and BHA was again used at a final concentration of 50 nM but on day 10 retinoic acid (RA; Sigma-Aldrich, R2625) was incorporated at a final concentration of 16 μM. After 13 days in culture, media replacement involved a 1:1 PBMCs-conditioned media to DMEM ratio and adding BHA 50 μM, RA 16 μM, Insulin Growth Factor-1 12.5 ng/ml (Peprotech, 100-11) and Neurotrophin-3 30 ng/ml (Peprotech, 450-03-100), all final concentrations. On day 17, cell culture media was not replaced, instead 25 mM Potassium chloride (KCL) was added (Sigma-Aldrich, P5405).

Cell viability was measured after cells were detached by incubating them for 4 min at 37°C with Trypsin-EDTA (0.05%), phenol red (ThermoFisher Scientific, 25300). After washing them, cells were stained with Trypan Blue (Trypan Blue Stain (0.4%), GIBCO, (1525061) following the manufacturers protocol. Live cells were enumerated by trypan blue exclusion. Results are shown as the ratio of the number of live cells compared to the number of original monocytes plated on day 0.

Pictures of cells were taken using a Nikon Eclipse Ti-S/L 100 inverted microscope equipped with a CoolSNAP Myo, 20 MHz, 2.8 Megapixel, 4.54 × 4.54 μm pixels camera and with a Nikon CFI Super fluor 20× DIC prism objective. Pictures were taken immediately after monocyte extraction and then at days 4, 7, 10 and 13 to establish a structural path to differentiation. Pictures were also taken around day 20 (days 19–22) when cells were already transdifferentiated. These pictures were used to establish differentiation rates. For one of the individuals in whom we collected two samples separated by several weeks, we observed lower cell concentration with the second sample. When measuring differentiation rates for this subject, we adjusted for low concentration by using the lowest concentration value on the first sample as a threshold. Any pictures from the second sample with a cell concentration lower than the threshold were not included in the differentiation rate analysis for this particular individual.

For treatment with dopamine, colchicine and control cells we analyzed transdifferentiated neuronal-like cells around day 20 from four healthy individuals (Table 1). Dopamine (Sigma-Aldrich, H8502-259) was diluted in ascorbic acid (Sigma-Aldrich, A4544) used at a concentration of 1 mg/ml. Colchicine was also from Sigma-Aldrich (C9754). At least 12 different fields were identified via a micro-ruled coverslip (Cellattice CLS5-25D, Nexcelom Bioscience) and pictures were taken before treatments. Cells were then treated with either dopamine 4 mM, colchicine 0.5 μM or without treatment (control). After incubating for 1 h at 37°C with 5% CO_2_ pressure, pictures of the exact same fields located via the micro-ruled coverslip were taken again. Only neuronal-like cells with at least one primary neurite longer than two times the soma size before treatment were traced. Only the longest primary and the longest secondary neurite for each cell traced were included in the analysis. Cells were traced manually using a semi-automated software called FIJI which is a plugin for ImageJ an open source image processing program. This same software was used for all structural analyses.

Human neurons were obtained from Innoprot (P10151) and cultured following the manufacturer instructions. We used Innoprot neuronal medium kit (P60157) and plastic plates were coated with Poly-L-Lysine, 1 mg/ml. Pictures of human neurons were taken after 5 days in culture with the same equipment used to take pictures of transdifferentiated neuronal-like cells. Human neurons were traced as described for neuronal-like cells. Only neurons with at least one primary neurite longer than two times the soma size were traced. Only the longest primary and the longest secondary neurite for each cell traced were included in the analysis. Neurons in which the exact ending of neurites was uncertain were not traced.

SH-SY5Y human neuroblastoma cells were a kind gift from Drs. Bernadette Allinquant and Christiane Rose (Psychiatry and Neurosciences Center, INSERM). U-373 MG human glioblastoma/astrocytoma cells were purchased from Merck (06081901). SH-SY5Y and U-373 MG cells were grown at 37°C with 5% CO_2_ pressure in DMEM, High Glucose, GlutaMAX (GIBCO, 61965059) supplemented with 10% FBS in which we added 100 U/mL penicillin; 100 mg/mL streptomycin. Once SH-SY5Y and U-373 MG cells reached confluency, cells were detached by incubating them with Trypsin-EDTA (0.05%), phenol red (ThermoFisher Scientific, 25300). SH-SY5Y cells used for structural comparison were treated with RA (16 μM) for 48 h. Neuroblastoma cells were traced as described for neuronal-like cells. Only cells with at least one primary neurite longer than two times the soma size were traced. Only the longest primary and the longest secondary neurite for each cell traced were included in the analysis. Cells in which the exact ending of neurites was uncertain were not traced.

Cancer cell lines Panc-1, Panc-02 and HT-29 were a kind gift from Drs. Gail Matters and Christopher McGovern (Penn State Hershey Medical Center, Department of Biochemistry and Molecular Biology, Hershey, PA, USA). These cell lines were grown at 37°C with 5% CO_2_ pressure in DMEM supplemented with 10% FBS in which we added 100 U/mL penicillin and 100 mg/mL streptomycin. Cells were detached as described above and used as controls in experiments of BrdU incorporation.

### Flow Cytometry

Cells were detached by incubating them for 4 min at 37°C with Trypsin-EDTA (0.05%), phenol red (ThermoFisher Scientific, 25300). After washing them, cells were treated with Blue Live/Dead Stain Kit (Life Technology, L23105) to exclude dead cells from analysis. Saturation was performed with either human group AB serum or Human BD Fc block (BD Pharmingen, 564220). For intracellular proteins, permeabilization was done with Triton X-100 0.2%. Cells were fixed with paraformaldehyde (PFA) 4%. Primary antibodies used were as follows: mouse IgM anti-human A2B5-APC (1/10, Miltenyi Biotec, 130-098-039), mouse IgG2a anti-human ß3-Tubulin-Alexa Fluor 647 (1/10, BD, 500394), mouse IgG2a anti-human CD14-Qdot-655 (1/20, Invitrogen, Q10056), mouse IgG2a anti-human CD34-PE (1/10, Miltenyi Biotec, 130-081-002), mouse IgG2b anti-human Dopamine Receptor 1-PE (1/5, BioLegend, 366404), mouse IgG2b anti-human GFAP-Alexa Fluor 488 (1/10, BD, 561449), chicken IgY anti-human Glutamate Decarboxylase 67 (1/20, Abcam, ab75712), mouse IgG1 anti-human Nestin-PerCp5.5 (1/10, BD, 561231), rabbit IgG anti-human PSD95-Alexa Fluor 488 (1/10, Abcam, ab195004), mouse IgG1 anti-human Sox2-Pacific Blue (1/10, Biolegend, 656112). Directly coupled extracellular and intracellular antibodies were respectively incubated during 20 min and 45 min on ice. Respective isotypes were incubated in parallel; mouse IgM-APC (1/10, Miltenyi, 130-099-085), mouse IgG2a-Alexa Fluor 647 (1/10, BD, 558053), mouse IgG2a-Qdot-655 (1/20, Invitrogen, Q10015), mouse IgG2a-PE (1/10, Miltenyi, 130-091-836), mouse IgG2b-Alexa Fluor 488 (1/2, BD, 558716), chicken IgY (1/2,000, AVES, #N-1010), mouse IgG1-PerCp5.5 (1/80, BD, 550795), rabbit IgG-Alexa Fluor 488 (1/10, Abcam, ab199091), mouse IgG1-Pacific Blue (1/10, Biolegend, 400151). Alexa Fluor 488 Goat anti-Rabbit (1/100, ThermoFisher Scientfic, A-11070) was used as secondary antibody and incubated 20 min on ice. Events were acquired using a fluorescence-activated cell sorting flow cytometer (FACS CANTO, Fortessa or LSR II) and analyzed using Diva (Version 6.1.1, BD Biosciences) followed by FlowJo (Version 10.1r7; TreeStar). Data acquisition and analysis were performed on the Cochin Cytometry and Immunobiology Facility.

### Immunofluorescence

Cells were washed with PBS, fixed with 4% PFA, treated with glycine 0.2 M, permeabilized with Triton X-100 at 0.2% and then blocked with 1% bovine serum albumin (BSA; Sigma-Aldrich) and 5% goat serum. Slides were mounted on Fluoromount-G (SouthernBiotech, 0100-01) and stained with 4’,6-Diamidino-2-Phenylindole, Dihydrochloride (DAPI; ThermoFisher Scientific, D1306). Immunolabeling was performed at 4°C overnight with the following primary antibodies: rabbit anti-MAP2 (1/25, Cell Signaling Technology, 4542), rabbit anti-Nestin (1/200, Millipore, AB5922), mouse anti-Neurofilament-M and Neurofilament-H (1/50, Millipore, MAB1592), mouse anti-CD14 (1/5, Immunotech, IOM2) and mouse anti-Tubulin (1/100, Zymed Laboratories, Invitrogen, 13-8000). Immunodetection was performed using species and subclass specific Alexa Fluor-405, Alexa Fluor-488 or Alexa Fluor-647 conjugated secondary antibodies (1/200, Life Technology). Antibodies were diluted in PBS containing 0.1% saponin and 0.3% BSA. Actin was stained with rhodamine phalloidin (1/200, Molecular Probes, Invitrogen, R415). Images were visualized under a wide-field microscope (Leica DMI 6000) equipped with a Micro MAX-1300YHS camera using an HCX PL APO 60X oil objective (Princeton Instruments). Images were acquired using Metamorph Software (Version 7.1.3; Molecular Devices).

### Single Cell RNA-Sequencing and Primary Data Processing

We utilized microfluidic single cell capture and single cell mRNA sequencing technologies to explore genome wide gene expression in 17 cells exposed to our transdifferentiation protocol. Fluidigm’s C1™ Single-Cell Autoprep System (C1) allows fully automated capture of up to 96 single cells and subsequent cDNA synthesis to then perform qPCR or RNA-sequencing. The cell suspension was loaded on the C1 by using an integrated fluidic circuit (IFC) chip which allows capturing a single cell per well. After optical confirmation of cell number at each capture site on the chip, the cells were processed for in-line cell lysis, reverse transcription and cDNA amplification steps. The resulting cDNA was subjected to a sequencing library using Illumina’s Nextera XT library preparation kit. The Rapid mode of Illumina HiSeq 2500 was used to generate sequencing reads of sufficient depth (about 3 million of sequencing reads) per each cell. De-multiplexed sequencing reads passed the default quality filtering of illumina casava pipeline (v1.8) and were then exposed to further quality trimmed/filtered using FASTX-Toolkit (v.0.0.13). The filtered reads were aligned to the most recent reference genome (hg38) using Tophat (v2.0.9; Trapnell et al., 2009) by allowing up to two mismatches. After normalization was performed via the median of the geometric means of fragment counts across all libraries, Fragments Per Kilobase per Million (FPKM) mapped reads values were calculated using Cuffdiff tool which is available in Cufflinks version 2.2.1 (Trapnell et al., 2010).

### Quantitative Real Time Polymerase Chain Reaction

qRT-PCR was performed as described previously (Panikashvili et al., 2006). Briefly, cells were detached from the plates using trypsin, and RNA was isolated using the Nucleospin RNA kit (Macherey-Nagel, Dren, Germany) or the RNeasy^®^ mini-kit (Qiagen, Hilden, Germany). The concentration of total RNA was assessed by NanoDrop™ (Applied Biosystems, Foster City, CA, USA) and adjusted to the same concentrations among samples. We used 125 ng aliquots of RNA to synthesize first strand cDNA using a High Capacity Reverse Transcriptase (Applied Biosystems, Foster City, CA, USA), with cycles completed in the Eppendorf Mastercycler Gradient (Hamburg, Germany) or the RT^2^ First Strand Kit (Qiagen, Hilden, Germany) that includes a DNase treatment. Experiments were performed in triplicates for quantitative PCR analysis using the Taqman Fast Advanced assay (ThermoFisher, Waltham, MA, USA) or the RT^2^ SYBR Green Mastermixes (Qiagen, Hilden, Germany) and using specific primers for PPIA, glutamic acid decarboxylase gene 1 and 2 (GAD1 and GAD2) (ThermoFisher, Waltham, MA, USA), GFAP (PPH02408F), MAP2 (PPH02419A), Nestin (PPH02388A), PDGFRα (PPH00219C), S100β (PPH02472F), Sox10 (PPH02458C) and ß3-Tubulin (PPH02607A, Qiagen, Hilden, Germany). Quantitative PCR were performed with an ABI 7900HT PCR system (Applied Biosystems, Foster City, CA, USA), or a LC480 light cycler (Roche, Basel, Switzerland). Ct numbers were calculated for both reference gene GAPDH and target genes with auto-baseline and auto-threshold. 2^−ΔΔCt^ was used to determine the fold increase. Data were collected and analyzed using the ExpressionSuite v1.1 software (ThermoFisher, Waltham, MA, USA) or LightCycler^®^ 480 (Roche, Basel, Switzerland).

### Electrophysiology

Blood circulating monocytes recuperated from leucoreduction filters from five different healthy individuals were used for recordings after transdifferentiation with our protocol. Recorded neuronal-like cells were continuously perfused at a rate of 2 ml/min throughout the experiment with gassed (95% O_2_, 5% CO_2_) artificial cerebrospinal fluid used as an external solution, containing 124 mM NaCl, 3 mM KCl, 2 mM CaCl_2_, 1 mM MgSO_4_, 1.25 mM NaH_2_PO_4_, 26 mM NaHCO_3_, 10 mM glucose (with pH 7.3, 300–310 mOsm/L). The patch pipettes were made of thin-walled borosilicate glass capillaries with a BB-CH horizontal pipette puller (Mecanex, Geneva, Swiss). The tip resistance of the recording pipettes was 4–6 MΩ. To study action potentials (APs), recording pipettes were filled with 140 mM K-Gluconate, 3 mM EGTA, 1 mM MgCl2, 10 mM HEPES, 2 mM ATP-Mg (with pH 7.3, 290–300 mOsm/L). After a gigaohm seal and whole-cell access was achieved, electrical activity was recorded using an Axopatch 1D amplifier (Axon Instruments, Union City, CA, USA), digitized using a Digidata 1200 interface (Axon Instruments). Delivery of command voltages and other analyses were driven by the pClamp 6 software from Axon Instruments (Clampex 6.0.4 for evoked responses and Fetchex 6.0.4 for spontaneous activities). Recordings were performed at room temperature (20–25°C). Cell input resistance and membrane capacitance transients were monitored during the entire recording.

### Cell Proliferation

Cell proliferation was measured via BrdU incorporation according to the manufacturer’s instructions (Calbiochem, QIA58) and based on absorbance at 450 nm–540 nm. Each experiment was done in triplicates with cells from six healthy individuals (Table 1). Human monocytes were cultured on fibronectin-coated 96-well plates for either 7 days or 8–10 days with one to two treatments of 100 nM BHA. Each well contained 80,000–130,000 cells. Background wells consisted of human monocytes cultured under the exact same conditions and at the same concentration but with no BrdU treatment. Our positive control and its background were cancer cell lines (Panc-1, Panc-02, or HT-29 cells) cultured at roughly the same concentration as monocytes. For the analysis, we obtained a reference readout and a raw readout for every well. We subtracted the reference readout from the raw readouts for test, background, and positive control wells then averaged the adjusted readouts for each type of well within each experiment. We compared transdifferentiation cultures with or without BrdU at day 7 or days 8–10 using the Mann-Whitney test.

### Statistical Analysis

Statistics were performed using medians and maximal and minimal values for small number of samples, mean and SEM for *n* > 30. A non-parametric Mann-Whitney test was used to make pairwise comparisons. ANOVA followed by Tukey’s post-test was used to make multiple comparisons. The analysis was done using Graph Pad Prism Software version 6.0f. *P* values lower than 0.05 were considered significant.

## Results

### Surface Markers, Structural Stages and BrdU Incorporation Through Early Stages of Differentiation

**Figure 1:**
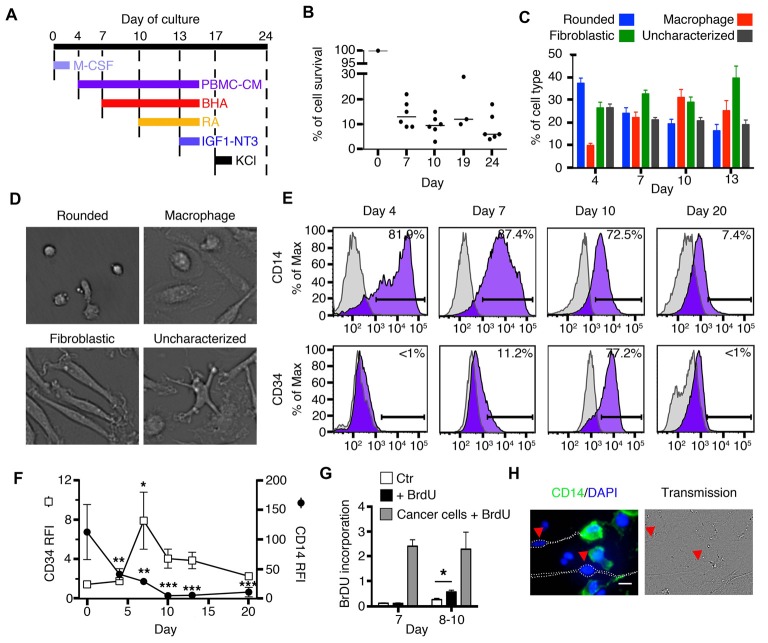
Early stages of transdifferentiation of human circulating monocytes into neural-like cells. **(A)** Culture outline. M-CSF was added on day 0. Butylated hydroxyanisole (BHA) was added on days 7, 10 and 13, retinoic acid (RA) on days 10 and 13, IGF1 and NT3 on day 13 and Potassium chloride (KCL) on day 17. Peripheral blood mononuclear cells (PBMCs)-conditioned autologous medium was added on days 4, 7, 10 and 13. **(B)** Global cell yield compared to the initial number of monocytes plated on day 0. Cells were trypsinized and live cells were enumerated by trypan blue exclusion. Results are shown as the ratio of the number of live cells compared to the number of original monocytes plated on day 0. Bars represent medians. *n* = 3–6 donors for each time point. **(C)** Bar graphs showing variations in the percentage of each cell type characterized by morphology during the first 13 days of transdifferentiation. Each colored bar corresponds to one of the four different cell types: rounded cells, macrophages, fibroblastic cells and uncharacterized cells. *n* = 15 donors (22,282 cells were characterized). **(D)** Representative pictures of the four different morphologies characterized during transdifferentiation from day 4 until day 13: rounded cells, macrophages, fibroblastic cells and uncharacterized cells. **(E)** Flow cytometric diagrams showing expression of CD14 and CD34 on days 4, 7, 10 and 20. Purple histograms represent specific labeling for either CD14 or CD34. Light gray histograms represent control isotypic labeling. **(F)** Relative Fluorescence Intensities (RFI) of CD14 and CD34 peaks found by flow cytometry along the culture. RFI are calculated as the ratio between the median fluorescence intensity of specifically labeled events (purple in E) over the median fluorescence intensity of control-labeled events (light gray in E). This allows to control for cellular autofluorescence changes along the culture. Differences compared to expression at day 0 were analyzed by One-way ANOVA followed by Tukey’s post-test. Results are expressed as mean ± SEM. *P < 0.05, **P < 0.01, ***P < 0.001. *n* = 5 at day 0, 5 at day 4, 9 at days 7, 10 and 13, 4 at day 20. **(G)** Bar graphs showing BrdU incorporation. White bars correspond to monocytes which were used as negative control (Ctr). Black bars (+BrdU) correspond to monocytes cultured for either 7 days (on the left) and treated with BrdU or monocytes cultured for 8–10 days (on the right) and also treated with BrdU. Gray bars correspond to cancer cell lines (either Panc-1, Panc-02, or HT-29 cells) treated with BrdU. We compared monocytes with or without BrdU at day 7 or days 8–10 using the Mann-Whitney test. *P < 0.05. *n* = 2 for day 7 and *n* = 4 for days 8–10. **(H)** Immunofluorescence (IF) and phase contrast images of cells transdifferentiated for 20 days. Cells expressing CD14 are labeled in green and counterstained with 4′,6-diamidino-2-phenylindole, dihydrochloride (DAPI) to show nuclear DNA in blue. Arrows point to cells with a neuronal phenotype in which CD14 expression is absent. Scale bar = 20 μm.

A transdifferentiation protocol from blood monocytes toward neuronal differentiation, was set up as outlined in Figure 1A. CD14+ monocytes purified from peripheral blood were cultured on fibronectin-coated flasks or culture wells using M-CSF. The culture medium was renewed by using PBMC-conditioned medium from the same donor on days 4, 7, 10 and 13. BHA was added, as a neuronal induction factor on days 7, 10 and 13 (Woodbury et al., 2000). RA was added as a neuronal-differentiation factor on days 10 and 13. Also on day 13, Neurotrophin-3 (NT-3) was added as a neuronal-differentiation factor due to its effects during neuronal development (Bellon et al., 2011), while Insulin-like growth factor 1 (IGF-1) was given due to its ability to regulate developing brain glucose metabolism (Rivers et al., 2008). KCl was added on day 17 to promote neuronal differentiation (He et al., 2011). The global median cell yield compared to the initial number of monocytes plated on day 0 was 13% (min 9−max 22%, *n* = 6) at day 7, and 10% (min 3−max 15%, *n* = 6) at day 10, 12% (min 10−max 29%) at day 19 and 6% (min 4−max 18%) at day 24 (Figure 1B).

In order to provide researchers with the ability to anticipate a structural path to transdifferentiation, we characterized 22,282 cells from 15 healthy individuals during the early stages of differentiation (days 4, 7, 10 and 13). The structural stages that monocytes underwent through the differentiation process were identified by pictures taken on days when media were changed, namely; days 4, 7, 10 and 13 (Figures 1C,D). We also studied the expression of two surface markers, CD14 and CD34, by FACS, Figures 1E,F). CD14 is a receptor abundantly expressed on monocytes and macrophages. On the other hand, CD34 is a surface marker for hematopoietic stem cells (Aghebati Maleki et al., 2014) and is not found on either monocytes or macrophages.

Immediately after magnetic isolation from fresh blood, monocytes in culture appeared as rounded cells of ~17 μm diameter, expressing high levels of CD14 and no CD34. After 4 days in culture, 37 ± 3% of the cells were still rounded, 9.8 ± 0.9% appeared as standard macrophages, which have a clearly identifiable nucleus and a flat extended, usually rounded cytoplasm (Figure 1D). Twenty six ± 3% of the cells took a fibroblastic shape while the remaining 26.5 ± 2% could not be characterized as either rounded, fibroblastic or standard macrophages. The percentages of CD14+ and CD34+ cells remained unchanged. By day 7, rounded cell percentages decreased to 24 ± 3%, macrophages slightly increased to 22 ± 3%, fibroblastic cells reached 33 ± 2%, while 21 ± 1% of the cells were uncharacterized. At this stage, cells continued to express CD14 but with lower intensity than before, and in contrast to previous days, a few cells became positive for CD34. By day 10, rounded cells dropped to only 19 ± 2%, macrophages increased to 31 ± 4%, while fibroblastic cells remained stable at 29 ± 2%. Some cells continued to express CD34 and most cells remained positive for CD14 but with dimmer intensity than before. At day 13, the changes were similar to those found on day 10 (rounded cells: 16 ± 3%, macrophages: 25 ± 5% and cells with fibroblastic shape: 40 ± 5% (Figure 1C).

Proliferation was tested using Bromodeoxyuridine (BrdU) incorporation. At day 7, when CD34 was first expressed, these cells did not incorporate BrdU; however, by day 10 and as early as day 8, there was a statistically significant increase in BrdU incorporation when compared to control cells cultured without BrdU (Figure 1G). Cancer cell lines, used as positive controls, proliferated at a much greater rate than transdifferentiating cells.

After 20 days in culture, we identified a population of cells that acquired a neuronal morphology. These cells had a well-delineated soma and long thin neurites (Figure 1H). Also by day 20, there was a decrease in the expression of CD14, while CD34 was no longer present (Figures 1E,F). Immunofluorescence (IF) revealed that cells with a neuronal phenotype no longer expressed CD14 (or its expression was very low), while cells without a neuronal phenotype generally remained positive for this monocytic surface marker (Figure 1H). Therefore, some cells at day 20 lost their monocyte/macrophage morphology and the CD14 marker and acquired a neuronal morphology.

### Expression of Neuroprogenitor and Neuronal Markers and Neuronal Type Characterization

**Figure 2:**
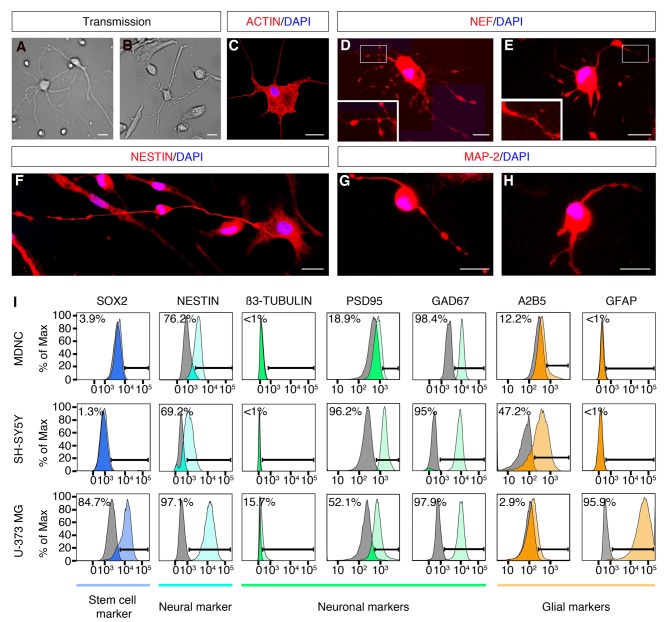
Expression of neuronal markers in human circulating monocytes transdifferentiated into neural-like cells. **(A)** Light microscopy photograph of human developing neurons (HDN) in culture for 5 days (20× original magnification). **(B)** Light microscopy photograph of transdifferentiated cells after 20 days in culture (20× original magnification). **(C)** IF photograph of a transdifferentiated cell with neuronal phenotype labeled with rhodamine phalloidin in red and with DAPI in blue for nuclear DNA (60× magnification). **(D,E)** IF photographs of transdifferentiated neuronal-like cells labeled with anti-neurofilament (NEF) antibodies in red and with DAPI in blue (60× magnification). Boxes present additional magnification of brighter spots inside neurites. **(F)** IF photographs of transdifferentiated neuronal-like cells labeled with anti-Nestin antibodies in red and with DAPI in blue for nuclear DNA (60× magnification). **(G,H)** IF photographs of transdifferentiated neuronal-like cells labeled with anti-MAP-2 antibodies in red and with DAPI in blue for nuclear DNA (60× magnification). Scale bars = 20 μm. **(I)** Flow cytometric diagrams showing expression of Sox2, Nestin, ß3-tubulin, PSD95, GAD67, A2B5 and GFAP on monocyte-derived-neuronal-like cells (MDNCs), SH-SY5Y human neuroblastoma cells (without RA treatment) and U-373 MG human glioblastoma/astrocytoma cells. Colored histograms represent specific labeling for each protein. Light gray histograms represent control isotypic labeling.

The morphology of the cells obtained with our transdifferentiation protocol was compared to that of human neurons (Figure 2A). In some instances, transdifferentiated cells extended only one neurite, but most commonly they were bipolar, although multipolar cells were also observed (Figures 2B,C).

In order to determine whether the population of cells that acquired a neuronal morphology expressed neuroprogenitor and/or neuronal markers, we performed IF, flow cytometry, qRT-PCR and single cell mRNA sequencing. IF demonstrated that cells with a neuronal morphology obtained at day 20 expressed the neuroprogenitor marker Nestin and neuronal markers such as; Neurofilament (NEF) and Microtubule-associated protein-2 (MAP-2; Figures 2D–H). The cellular distribution of these proteins was similar to what has been reported during differentiation of neurons and neuronal cell lines in culture (Cáceres et al., 1986; Tohyama et al., 1993; Shea and Beermann, 1994). Nestin, Neurofilament and MAP-2 were present in the cellular soma as well as along neurites of neuronal-like cells (Figures 2D–H). Note that some IF show brighter spots along neurites (Figures 2D,E,G). It is possible that these brighter spots are varicosities, a sign of dendritic injury in degenerating neurons. Also note that those brighter spots were not always present (Figures 2C,F,H).

We further performed flow cytometric labeling of characteristic neuroprogenitor, neuronal, glial and stem cell proteins on neuronal-like cells obtained at days 19–24, compared with SH-SY5Y neuroblastoma and U373 MG astrocytoma/glioblastoma cell lines (Figure 2I). This method is sensitive, specific and quantitative, as it shows the percentage of cells as a function of their specific labeling intensities (colored histograms), compared to control labeling (gray histograms) with a non-specific antibody or antiserum with the same isotype as the specific antibody. Cells treated with our transdifferentiation protocol did not express Sox2, a neuronal progenitor stem cell marker, which is expressed in U373 MG cells (Figure 2I). Neuronal-like cells expressed Nestin, a protein expressed throughout the development from neuronal progenitors to mature neurons (median: 81% of the cells, min 2% max 100%, *n* = 6). They did not express β3-tubulin, similarly to SH-SY55 cells. They clearly expressed the post-synaptic density protein 95 (PSD95; median: 36% of the cells, min 0% max 100%, *n* = 8). The GABAergic marker GAD-67, was tested in one experiment where it was clearly expressed. They weakly expressed the ganglioside A2B5, a neuronal and glial progenitor cell marker. Conversely, they did not express GFAP, a marker for astrocytes expressed in U373 MG cells. Therefore, the flow cytometric expression profile of the cells treated with our transdifferentiation protocol was comparable to that of the SH-SY5Y cell line.

**Figure 3:**
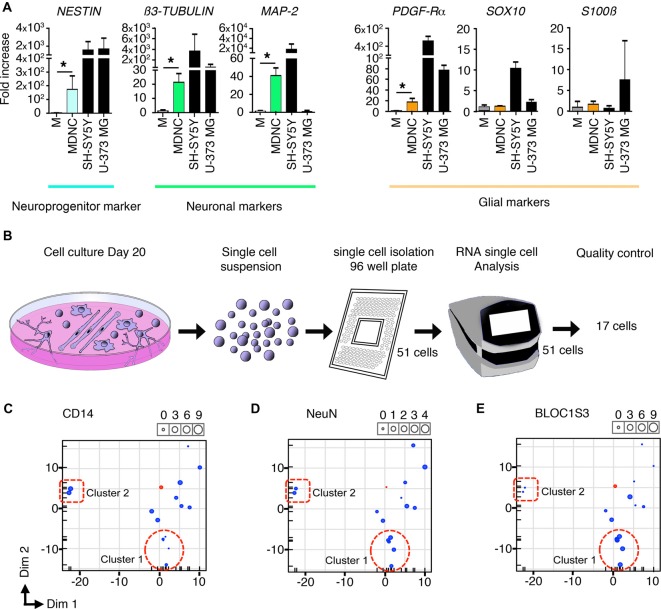
Transcriptomic analysis of human circulating monocytes transdifferentiated into neuroprogenitor-like cells. **(A)** Expression of mRNA coding for neuroprogenitor, neuronal or glial genes. MDNCs after 20 days in culture were compared to monocytes (M) from the same donor at day 0. Transcription was assessed by qRT-PCR. Relative quantification over *GADPH*, *n* = 4 experiments for *Nestin*, 5 for β3-*Tubulin*, 4 for *MAP-2*, 5 for *PDGFR-α*, 4 for *Sox10*, 5 for *GFAP* and 5 for *S100β*. Bars represent mean ± SEM. Comparisons were performed using the Mann-Whitney test. *P < 0.05. **(B)** Schematic representation of the single cell mRNA sequencing procedure. From left to right, representation of monocytes cultured for 20 days and treated with our transdifferentiation protocol, these same cells in suspension, isolation of 51 cells into a 96-well plate, C1 machine into which 51 cells were subjected to RNA analysis, finally the 17 cells that passed the quality control for single cell mRNA sequencing analysis. **(C–E)** Three principal component analysis (PCA) plots one for each of the following genes; CD14, which is highly expressed in monocytes and macrophages **(C)** NeuN, a commonly used neuronal marker **(D)** and BLOC1S3, a gene implicated in synaptic transmission **(E)** Every dot inside the plot represents a single cell. The size of the dot is proportional to the level of expression of each gene. Blue dots represent the 17 cells treated with our transdifferentiation protocol. Pink dots correspond to THP-1 a monocytic cell line to serve as control. Two clusters of cells can be identified. Cluster 1 outlined with a dotted circle, shows cells expressing very low levels of CD14, moderate expression of NeuN and high levels of BLOC1S3. A second cluster of cells outlined with a dotted square, presents very high levels of CD14, lower expression of NeuN and very low levels of BLOC1S3.

We also performed a transcriptomic analysis by qRT-PCR (Figure 3A). Compared to starting monocytes for each experiment at day 0, cells treated with our transdifferentiation protocol expressed significantly more mRNA coding for Nestin, the neuronal proteins β3-tubulin and MAP-2, and also for PDGF-Rα, a protein strongly expressed in oligodendrocytes but also found in some neuronal progenitors (Rivers et al., 2008). In contrast, they did not express mRNA exclusively associated with either oligodendrocytes or astroglia such as; *Sox10*, *GFAP* or *S100β*. Their transcriptomic expression profile analyzed by qRT-PCR was comparable to that of the SH-SY5Y cell line. In addition, cells treated with our transdifferentiation protocol expressed the *GAD 1*, which encodes for GAD-67 (Supplementary Figure S1).

Finally, we performed single cell mRNA sequencing on 17 cells treated with our transdifferentiation protocol (Figure 3B) in order to determine whether the neuronal-like cells obtained were glutamatergic, GABAergic, dopaminergic, serotoninergic, cholinergic or motor neurons. Based on the expression of genes specific for these neuronal types (Supplementary Table S1) it is clear that our protocol delivered neuronal-like cells and expressed genes associated with glutamatergic, GABAergic, dopaminergic and serotoninergic neurons. Although their pattern of expression (Supplementary Table S1) cannot allow to definitely categorize them, some cells tend to be more serotoninergic (cell c) or glutamatergic (cells h, i, k, n, o). There was no expression of cholinergic or motor neurons genes. This expression pattern is similar to that shown by SH-SY5Y cells as shown in Supplementary Table S1.

The pattern of expression of these cells was further analyzed using Principal Component Analysis (PCA). FACS results indicate that through the transdifferentiation process cells decreased their expression of CD14 (Figures 1E,F) resulting in low or undetectable expression of CD14 in cells acquiring a neuronal morphology (Figure 1H). At the same time, neuronal-like cells increased their expression of neuronal markers (Figures 2D–H). To determine whether single cell mRNA sequencing experiments support this finding, we developed three PCAs, each based on the expression of either *CD14* (Figure 3C), which is highly expressed in monocytes and macrophages, *NeuN*, a commonly used neuronal marker (Figure 3D) or *BLOC1S3*, a gene implicated in synaptic transmission (Gokhale et al., 2016; Figure 3E). Two different clusters of cells were identified. Cluster 1, outlined with a dotted circle, comprised cells expressing very low levels of *CD14*, moderate expression of *NeuN* and high levels of *BLOC1S3* (Figures 3C–E). Cluster 2, outlined with a dotted square, presented very high levels of *CD14*, lower levels of *NeuN* and very low levels of *BLOC1S3* (Figures 3C–E). These results support that cells with increased expression of neuronal genes, decreased their *CD14* levels.

### Electrophysiological Recordings of Neuronal-Like Cells

**Figure 4:**
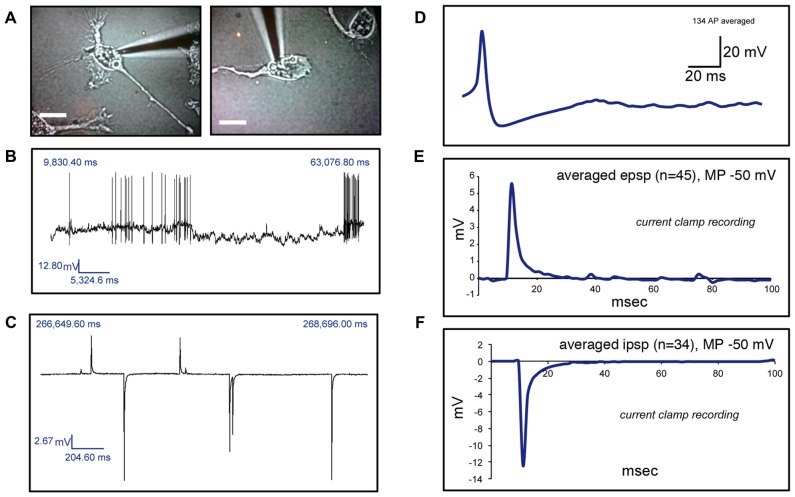
Electrophysiological recordings of human circulating monocytes transdifferentiated into neural-like cells. **(A)** Light microscopy photographs of two representative neuronal-like cells in which electrical activity was found. Scale bars = 20 μm. **(B)** Recordings under current clamp conditions showing spontaneous action potentials (APs) on a transdifferentiated neuronal-like cell. **(C)** Recordings under current clamp conditions showing spontaneous excitatory postsynaptic potentials (EPSPs) and inhibitory postsynaptic potentials (IPSPs) on a transdifferentiated neuronal-like cell. **(D)** Average of 134 APs present in one transdifferentiated neuronal-like cell. **(E)** Average of 45 EPSPs present in one transdifferentiated neuronal-like cell. **(F)** Average of 34 IPSPs present in the same transdifferentiated cell shown in **(E)**. Of note, over 200 recordings were obtained from 11 cells from five different healthy individuals.

We then investigated whether cells with a neuronal phenotype presented electrical activity under current clamp conditions. Pictures of two representative cells in which electrical activity was found are shown in Figure 4A. Transdifferentiated cells obtained from leucoreduction filters from five different healthy individuals were used for recordings. Twenty cells were tested. Three cells provided inconclusive results. On six cells we did not find any electrical activity while 11 cells showed electrical activity (Table 2). Over 200 recordings were obtained. We observed spontaneous APs (Figure 4B) as well as spontaneous excitatory postsynaptic potentials (EPSPs) and inhibitory postsynaptic potentials (IPSPs; Figure 4C). The average frequency for APs encountered was 0.08 Hz (range 0.01–0.14 Hz) and the average mean amplitude was 43.50 mV (range 37–50 mV). For EPSPs, the average frequency was 0.27 Hz (range 0.005–0.9 Hz) and the average mean amplitude was 12.22 mV (range 3–32 mV). In the case of IPSPs, the average frequency was 0.40 Hz (range 0.003–0.87 Hz) and the average mean amplitude was −8.80 mV (range −20 to −2 mV). The average membrane resting potential was −51.0 mV (range −34 to −65 mV). Note that only cells with a neuronal phenotype generated electrical activity whereas cells with other phenotypes did not.

**Table 2:** Summary of electrophysiological recordings from monocyte-derived-neuronal-like cells (MDNCs).

Action potentials					
Frequency Hz			Mean Amplitude mV		
0.14	Average	0.08	37	Average	43.5
0.01	Standard		50	Standard	
	error	0.07		error	6.5
**Excitatory Postsynaptic Potentials**					
0.005			17		
0.17			4		
0.01			9		
0.005			18		
0.86	Average	0.27	6	Average	12.2
0.9	Standard		3	Standard	
	error	0.13		error	3.1
0.47			5		
0.005			16		
0.009			32		

Inhibitory Postsynaptic Potentials*					
0.65			−12		
0.45			−5		
0.87	Average	0.40	−20	Average	−8.80
0.003	Standard		−5	Standard	
	error	0.17		error	3.25
0.007			−2		

Each row corresponds to one cell and each color corresponds to a different donor. *IPSPs were always found in cells that also presented EPSPs, hence; IPSP rows should not count as individual cells. The total number of cells with electrical activity was 11.

### Structural Stages Leading to Neuronal Differentiation and Differentiation Efficiency

In 2002, Dotti and DaSilva identified several clearly defined structural stages that neurons undergo during the transformation from rounded cells to cells with complex forms *in vitro* (da Silva and Dotti, 2002). These stages include: (1) rounded cells; (2) unipolar or bipolar cells with very short extensions; (3) stellate cells with thick extensions and flat soma; and (4) cells with a well-defined soma and long thin extensions that can be unipolar, bipolar or multipolar (da Silva and Dotti, 2002). In order to determine whether our cells progress through similar structural stages, we followed a group of cells during the last days of differentiation with serial photographies. Once the transdifferentiation process was completed, we identified cells that developed a neuronal morphology and retrospectively followed the structural stages that these exact same cells underwent. We were able to delineate the structural path to neuronal morphology of five cells from two different individuals and these cells followed similar structural stages as those described for neurons (Supplementary Figure S2). It is important to note that transdifferentiated cells, just as neurons early in development, presented a high degree of structural plasticity.

In a first approximation to establish the differentiation efficiency obtained with our protocol, we characterized morphologically 38,819 cells from 15 healthy individuals (blood collected in EDTA tubes) between days 19–22 into either cells with or without a neuronal morphology (a well-delineated soma and at least one long thin neurite longer than two times the soma size). This approach showed a differentiation efficiency of 11.9 ± 1.4% (mean ± SEM). We then explored whether methods of blood collection impacted differentiation efficiency and found that 5,063 cells obtained from three healthy donors isolated from leucoreduction filters evidenced a 12.8 ± 0.32% (mean ± SEM) of cells with neuronal morphology. Another strategy to determine differentiation efficiency was to analyze the expression of PSD95 by flow cytometry (Figure 2I). PSD95 was selected because it is a synaptic marker and therefore found in mature neurons. PSD95 was expressed in 36% of the cells (min 0% max 100%, *n* = 8) measured at day 20.

### Structural Responses to Colchicine and Dopamine

Before studying structural responses to colchicine and dopamine, we compared transdifferentiated neuronal-like cells (MDNCs) with SH-SY5Y neuroblastoma cells and with human developing neurons (HDN) after 5 days in culture. Four structural parameters were used for this comparison; primary neurite length, secondary neurite length and the number of primary and secondary neurites per cell (Supplementary Figure S3). The length of secondary neurites was practically the same between the three cell types (21 ± 0.7 μm for MDNCs, 21.5 ± 1 μm for SH-SY5Y and 18.6 ± 2 μm for HDN, mean ± SEM). Primary neurite length was comparable between the three types of cells but there were statistical differences. HDN had longer primary neurites (99 ± 3.8 μm, mean ± SEM) than the other two cell types and neuroblastoma cells had the shortest (93 ± 1.7 μm for MDNCs and 76 ± 1.8 μm for SH-SY5Y, mean ± SEM). Number of primary (5 ± 0.1, mean ± SEM) and secondary neurites (9 ± 0.4, mean ± SEM) was significantly higher in transdifferentiated neuronal-like cells, while SH-SY5Y had the lowest number of primary neurites (3.7 ± 0.2 for HDN and 3.6 ± 0.1 for SH-SY5Y, mean ± SEM) and HDN had the lowest number of secondary neurites (2.8 ± 0.2 for SH-SY5Y and 1 ± 0.1 for HDN, mean ± SEM). Of note, if cultured for a longer time, human neurons continue to extend neuronal processes and their arborizations become more complex, whereas neuroblastoma cells and transdifferentiated neuronal-like cells remain stable. But after 5 days in culture, HDN have similar structural characteristics to neuroblastoma cells and transdifferentiated neuronal-like cells.

**Figure 5:**
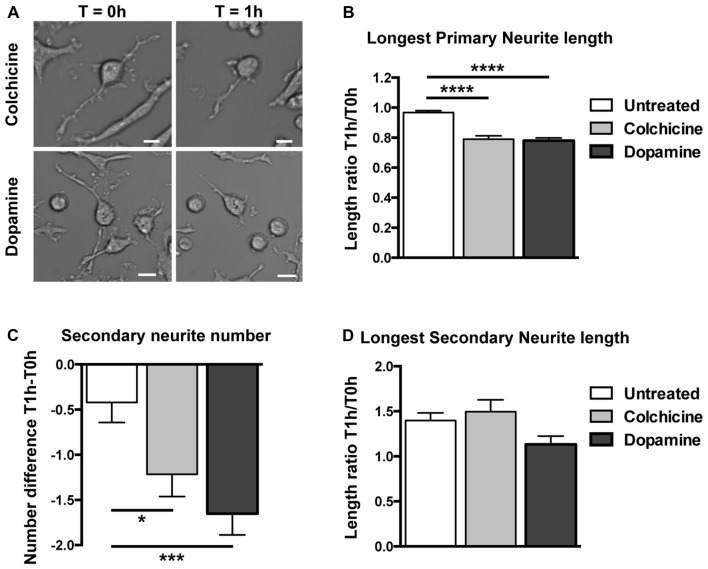
Structural responses to dopamine and colchicine exposure on human circulating monocytes transdifferentiated into neural-like cells. **(A)** Light microscopy photographs before and after treatment. The same cell was photographed before (*T* = 0 h) and after (*T* = 1 h) treatment with colchicine 0.5 μM (top), and another cell before and after treatment with dopamine 4 mM (bottom). Scale bars = 20 μm. **(B)** Bar graph showing a decrease in length of the longest primary neurite of transdifferentiated neuronal-like cells before and after different treatments. Data are expressed as ratios between the length of the longest primary neurite of each cell measured before (T0 h) and after (T1 h) treatment. The mean total length of the primary neurites at *T* = 0 h was 70 ± 30 μm. **(C)** Bar graph showing the number of secondary neurites of transdifferentiated neuronal-like cells. Data are expressed as the difference between the number of secondary neurites before (T0 h) or after (T1 h) treatment. The mean number of secondary neurites at *T* = 0 h was 6.0 ± 5.3. **(D)** Bar graph showing length of the longest secondary neurite on transdifferentiated neuronal-like cells before and after different treatments. Data are expressed as described in **(B)**. The mean total length of the secondary neurites at *T* = 0 h was 12 ± 8 μm. Statistics are given mean ± SEM. *n* = 256 cells. Differences were assessed by one-way ANOVA. *P < 0.05, ***P < 0.001, ****P < 0.0001.

To determine if these neuronal-like cells mimic neuronal responses to colchicine and dopamine, we challenged them with these compounds, separately. Minimal but statistically significant retraction of longest primary neurite and decrease in the number of secondary neurites was seen in untreated cells. This shrinkage is likely the reaction of cells being transported from the incubator to the microscope while being at room temperature (Figure 5A). To control for this shrinkage, we enumerated and measured the same cells at time T0 h before treatment and at time T1 h after treatment with either medium (untreated), colchicine or dopamine and calculated the neurites length ratios for each cell at T1 h over T0 h, and the difference in the numbers of secondary neurites at T1 h − T0 h (Figures 5B,C). Colchicine is well-known for its ability to elicit neurite retraction in neurons (Daniels, 1972) and in neuronal cell lines (Brat and Brimijoin, 1992) via microtubule depolymerization (Drubin et al., 1988). Just as neurons and neuronal cell lines, transdifferentiated neuronal-like cells incubated for 1 h with 0.5 μM of colchicine retracted their longest primary neurite (Figures 5A,B) and decreased the number of secondary neurites (Figures 5A,C) while their longest secondary neurite remained unchanged (Figure 5D). The concentration of colchicine needed to elicit such a response corresponded to those utilized in neurons (Daniels, 1972) and in neuronal cell lines (Brat and Brimijoin, 1992). It has also been consistently shown that neurons in culture retract their extensions when exposed to dopamine (Rodrigues and Dowling, 1990; Lieb et al., 1995; Reinoso et al., 1996). Likewise, our neuronal-like cells retracted their longest primary neurite (Figures 5A,B) and the number of secondary neurites also decreased (Figure 5C). The concentration of dopamine we utilized (4 mM) was higher than those reported for neurons but our incubation time was significantly shorter (see “Materials and Methods” section).

### Reproducibility of Results From Sequential Blood Samples From the Same Individual

**Figure 6:**
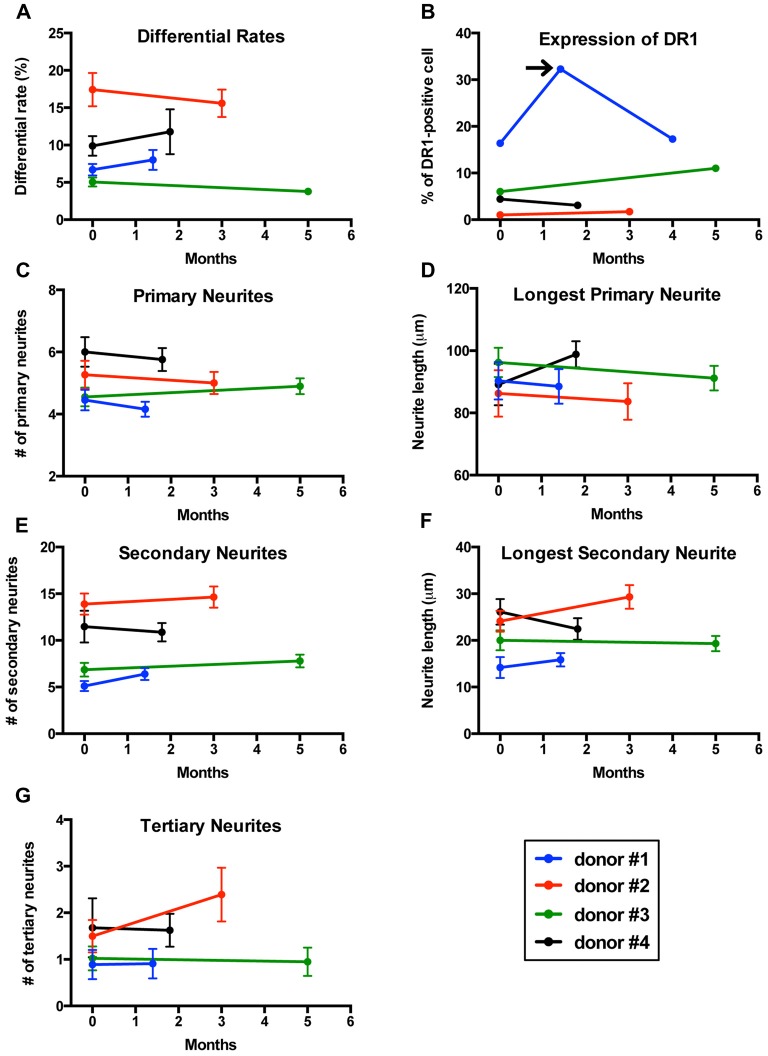
Reproducibility of results in sequential samples from the same donors. **(A)** Line graph showing differentiation efficiency assessed by neuronal morphology on two separate blood samples transdifferentiated into neuronal-like cells from four healthy men. Each line represents a different individual. **(B)** Line graph showing the percentage of cells expressing dopamine 1 receptor (DR1). Arrow points to a sample from one of the individuals that was not consistent with the other two samples. **(C)** Line graph showing the number of primary neurites. **(D)** Line graph showing length of the longest primary neurites. **(E)** Line graph showing the number of secondary neurites. **(F)** Line graph showing length of the longest secondary neurites. **(G)** Line graph showing the number of tertiary neurites.

If an *in vitro* model is to be used to compare cells from two different groups (patients vs. controls), it is essential that the model delivers reproducible results. To determine whether our method was reproducible, we collected two sequential blood samples from four healthy men to transdifferentiate their monocytes into neuronal-like cells and measured differentiation efficiency, length and number of neurites, as well as expression of dopamine 1 receptors (DR1). Blood samples were collected 1.5–5 months apart (Figure 6).

Differentiation percentages established via morphology remained consistent in this group of healthy donors (Figure 6A). For one of the individuals tested, cell concentration was significantly reduced on the second sample and therefore we had to adjust for this variation in concentration (see methods section for details). Expression of DR1 measured via flow cytometry also showed reproducible results in all individuals except for one patient who was sampled three times (Figure 6B), where only the first and third samples provided consistent results. We then analyzed the structure of neuronal-like cells. Several morphological parameters including longest primary neurite, number of primary neurites, longest secondary neurite, number of secondary neurites and number of tertiary neurites remained stable between the first and second sample in all four healthy donors (Figures 6C–G).

## Discussion

Despite progress, our understanding of psychiatric and neurological illnesses at a cellular level remains poor, due at least in part to the inability to access neurons directly from patients. The paradigms currently available such as IPSCs, BM-MSCs and ONCs are beginning to advance the field. But significant work remains, including the search for a noninvasive, inexpensive and rapid method to obtain neuronal-like cells with the capacity to deliver reproducible results.

Transdifferentiation is a promising alternative that has already shown some advantages over IPSCs. For instance, Mertens et al. (2015) demonstrated that direct conversion of human fibroblasts into neurons but not IPSCs-derived neurons, maintain donor age-dependent transcriptomic signatures and therefore are better suited to model human aging. The transdifferentiation potential of more accessible cell types has also been tested. Several research teams have shown that a fraction of human circulating monocytes have pluripotent properties (Kuwana et al., 2003; Zhao et al., 2003). Cultured under the right conditions, these cells were shown to express neuronal markers (Zhao et al., 2003; Romagnani et al., 2005; Porat et al., 2006) and even display structural resemblance to neurons (Kodama et al., 2006; Horschitz et al., 2010). But in order to incorporate transdifferentiation as a tool for translational medicine, it is necessary to provide comprehensive evidence that human circulating monocytes can be easily and consistently transdifferentiated into the neuronal lineage.

Here, we introduce a method that can fulfill these needs. However, this new protocol has its own set of limitations. Therefore, in the following lines, we contrast our results with the other three currently available paradigms in order to present advantages and disadvantages for each *in vitro* model.

In the protocol described here neuronal-like cells were obtained in 20 days from a standard blood sample. This differentiation time represents an advantage over IPSCs, for which the entire process could take months (isolation of fibroblasts, genetic transduction, IPSCs selection, direct differentiation; Dolmetsch and Geschwind, 2011; Borgmann-Winter et al., 2015; Petersen and Strappe, 2016) and is comparable to protocols available for BM-MSCs (Krabbe et al., 2005) and ONCs (Gomez et al., 2000). The final stage of IPSCs differentiation into neurons has recently evidenced significant advances, including the emergence of new protocols that deliver higher differentiation efficiency (Zhang et al., 2013; Goparaju et al., 2017), though some of the other IPSCs limitations remain. For instance, blood sampling is less invasive than the alternatives. Most protocols currently available for IPSCs require a skin biopsy. Access to BM-MSCs depends on a bone marrow aspirate while ONCs most commonly originate from an olfactory mucosa biopsy. These samplings involve consulting a specialist and entail physical discomfort together with risk for more serious complications. MDNCs are thus practical, offering the possibility to obtain neuronal-like cells relatively rapidly and via a standard blood sample. While time to differentiation and invasiveness are relevant factors to consider, there are other essential characteristics that need to be pondered, such as the production of mature neurons.

Only ONCs provide actual mature neurons (Borgmann-Winter et al., 2009), albeit not always, due to the heterogeneity of the olfactory epithelium (Féron et al., 1998; Gomez et al., 2000; Borgmann-Winter et al., 2015). The most common scenario is to encounter cells at different stages of maturation (Borgmann-Winter et al., 2015). Similarly, BM-MSCs deliver cells at varying degrees of differentiation (Krabbe et al., 2005). But there are also protocols available that can generate more homogeneous cultures from BM-MSCs (Nandy et al., 2014). Protocols utilizing IPSCs continue to progress in the degree of neuronal specification and level of maturation (Liu and Zhang, 2011; Wen et al., 2016). Therefore, IPSCs should be considered the most advanced paradigm to generate specific types of neurons (Wen et al., 2016). In contrast, MDNCs are in the very early stages of neuronal differentiation as they express a combination of neuroprogenitor and neuronal markers (Figures 2D–I). Their level of immaturity is evidenced by the expression pattern of some neuronal markers. For instance, β3-tubulin is only limited to mRNA expression (Figure 3A) and not found at the protein level (Figure 2I). Also consistent with their immaturity is their expression pattern that cannot quite categorize them into a single neuronal type (Supplementary Table S1). MDNCs express genes present in glutamatergic, GABAergic, dopaminergic and serotoninergic neurons, similarly to what is found in neuroblastoma cells (Supplementary Table S1). In fact, resemblance to neuroblastoma cells is not only limited to their gene expression pattern, the structure of MDNCs is also similar to that of neuroblastoma cells (Supplementary Figure S3). For researchers seeking to study mature neurons this is a disadvantage. For others, particularly those studying brain disorders considered neurodevelopmental illnesses such as autism and schizophrenia, it is an advantage, as this model provides a window into brain development.

A common question when neuronal-like cells are developed *in vitro* is whether they generate electrical activity. To our knowledge, this is the first report of electrical activity in MDNCs. It is important to note that, while we recorded spontaneous APs (Figure 4B), the most common findings were EPSPs and IPSPs (Figure 4C). We are currently working on establishing the type of channels present in these cells; thus, our understanding of the electrophysiological properties of MDNCs is only beginning. Comparably, the characterization of electrical activity displayed in ONCs is in its early stages (Benítez-King et al., 2011; Borgmann-Winter et al., 2015), whereas for BM-MSCs (Liu et al., 2012) and for IPSCs (Jiang et al., 2010) a more comprehensive account of their electric currents has been gained.

While electrical activity is a relevant feature, viability is an element often overlooked. For MDNCs, viability declines after 24 days in culture (Figure 1B). Hence, we perform most of our experiments between days 19 and 22. Similar limitations are seen with BM-MSCs (Deng et al., 2001; Rismanchi et al., 2003). On the other hand, it is rare to find reports on cell viability with ONCs and IPSCs. Perhaps, cell maintenance after differentiation is not an issue with these models; although, some authors have reported high rates of cell death under certain culture conditions with IPSCs (Wang et al., 2015). We are currently searching different approaches to prolong MDNCs survival, including co-culture with glial cells as well as the use of other additives and growth factors.

Another relevant characteristic of stem cells is their ability to proliferate. This ability provides researchers with the convenience of expanding and storing stem cells for future testing. IPSCs, BM-MSCs and ONCs can all be expanded and stored. MDNCs proliferate, but at a very slow rate (Figure 1G). We, therefore, have not expanded MDNCs. Instead, we find it easier to obtain a second blood sample whenever we require further testing of a particular individual. This, however, can be inconvenient and, in certain cases is not possible and thus is a limitation of MDNCs.

Differentiation efficiency is systematically calculated within the stem cell field. Unfortunately, approaches on how to estimate differentiation percentages vary tremendously. We measured MDNCs differentiation efficiency via cell morphology and through expression of PSD95. Our results indicate differentiation efficiencies of 12%–36%. Ranges of efficiency for the other three *in vitro* paradigms to place MDNCs in perspective are as follow. For BM-MSCs there are reports that range from 13% to 51% differentiated cells (Rismanchi et al., 2003). However, the protocol with the highest differentiation rates also elicits increased cell mortality (Rismanchi et al., 2003). ONCs reports of neuronal prevalence vary from 20% to 65% (Gomez et al., 2000; Benítez-King et al., 2011). The caveat here is that some authors reported only 66% success when attempting to grow olfactory biopsies (Gomez et al., 2000). This means that one third of the time researchers were unable to obtain olfactory neurons. IPSCs differentiation percentages fluctuate from 15% to 79%, but the variability here is not from different protocols or different individuals. Cell lines generated from the same fibroblasts originating from the same person provide inconsistent results (Hu et al., 2010; Carcamo-Orive et al., 2017). This variability is concerning since reproducible results are essential if the goal is to compare cells between patients and controls (Bellin et al., 2012). Not surprisingly, lack of reproducibility between samples from the same donor represents the main impediment for the clinical use of IPSCs (Fossati et al., 2016).

MDNCs provided relatively reproducible results for length and number of arborizations, differentiation percentages, as well as for expression of DR1 (Figure 6). These results, however; have to be considered preliminary as they only included men and the sample size is small. Also relevant, is to consider that while the expression of DR1 shows minimal variation over time, other proteins might behave differently and thus each protein considered for future analysis has to be deemed stable over time before comparing it between different cohorts. It is also important to keep in mind that MDNCs present variability between donors. Therefore, when MDNCs are used for comparisons between cohorts, it is necessary to stratify each group, at least based on differentiation efficiency in order to avoid biases. As mentioned, generating reproducible results with IPSCs has been difficult (Hu et al., 2010; Bellin et al., 2012; Fossati et al., 2016; Carcamo-Orive et al., 2017). We are not aware of any study demonstrating reproducible results with either BM-MSCs or ONCs and concerns have been raised about variations between samples with ONCs (Borgmann-Winter et al., 2015).

In summary, here we present a new protocol to transdifferentiate human circulating monocytes into neuronal-like cells that has been tested in 68 individuals in two different laboratories located in separate continents (Table 1). In addition, MDNCs are a practical and rapid method to generate neuronal-like cells as they can be obtained from a standard blood sample and in only 20 days. Because MDNCs are inexpensive and relatively rapid to obtain, researchers can study larger numbers of patients than what is currently seen when stem cells are used as translational research tools. More importantly, our results indicate MDNCs deliver reproducible results and therefore, opens the door for studying complex multigenetic illnesses such as schizophrenia and autism. But MDNCs also have disadvantages including: (1) resemblance to immature neurons; (2) viability declining after day 24 in culture; (3) slow proliferation rates; and (4) low differentiation efficiency. Despite these limitations, we are convinced that MDNCs are a valuable translational tool as they offer a new methodology to obtain neuronal-like cells directly from patients.

## Author Contributions

AB: planned and developed most experiments and wrote this manuscript. AW: developed experiments especially most of the flow cytometric and qRT-PCR experiments, designed most of the figures and participated in writing the manuscript. ARL: developed several experiments. MV: participated in the development of several experiments. SKY: developed experiments pertaining to electrophysiology. RG: analyzed the electrophysiology results. JM: developed several experiments. FM: participated in the planning of some experiments. PW: analyzed the experiments pertaining to structural stages during differentiation. LV: participated in the development of several experiments. JKY: participated in the planning of some experiments. YIK: developed the experiments and analysis of the single cell genotyping. GAC: participated in the development and planning of the experiments pertaining to single cell genotyping and helped with manuscript preparation. EB: developed and analyzed some experiments with neuroblastoma cells and human neurons. BC: contributed to the analyses of the single cell mRNAsequencing data. TMJ: participated in the planning of several experiments. MOK: participated in the planning of experiments, participant selection and in the development of this manuscript. VF: participated in the planning, development and analysis of several experiments and in the writing of this manuscript. AH: participated in the planning and interpretation of experiments and in the writing of this manuscript.

## Conflict of Interest Statement

AB, MOK, VF, TJ and AH are inventors of a patent filed in the USA and Europe under patent numbers US9932556 and EP2862926 by SATT IDF Innov on behalf of CNRS, INSERM and Université Paris Descartes. The remaining authors declare that the research was conducted in the absence of any commercial or financial relationships that could be construed as a potential conflict of interest.

## Supplementary Material

FIGURE S1 Glutamic Acid Decarboxylase (GAD) gene expression in human circulating monocytes transdifferentiated into neural-like cells. Bar graphs depicting expression of Glutamic Acid Decarboxylase 1 and 2 (GAD1 and GAD2) relative to Cyclophilin A (PPIA), which was used as a reference gene. GAD1 encodes for GAD67 and GAD2 encodes for GAD65. Both proteins are considered markers for GABAergic neurons. GAD1 was expressed in monocyte-derived-neuronal-like cells (MDNCs) but not evident in undifferentiated monocytes (**P* < 0.029). GAD2 is not expressed in either MDNCs or monocytes. These bars represent four experiments done by triplicate with cells from three different healthy subjects.

FIGURE S2 Structural stages leading to a neuronal morphology. These six light microscopy photographs correspond to the exact same transdifferentiated neuronal-like cell followed through the last days of differentiation. Each photograph corresponds to one of the structural stages that neurons undergo before acquiring their complex structure. These light microscopy photographs are representative of two separate experiments. Scale bars = 20 μm.

FIGURE S3 Structural comparison between human neurons, neuroblastoma cells and human circulating monocytes transdifferentiated into neural-like cells. Bar graphs showing several structural parameters for MDNCs, SH-SY5Y human neuroblastoma cells and human developing neurons (HDN) after 5 days in culture. **(A)** Bar graph showing the length in μm of the longest primary neurite of MDNCs, SH-SY5Y and HDN. **(B)** Bar graph showing the length in μm of the longest secondary neurite of MDNCs, SH-SY5Y and HDN. **(C)** Bar graph showing the number of primary neurites per cell on MDNCs, SH-SY5Y and HDN. **(D)** Bar graph showing the number of secondary neurites per cell on MDNCs, SH-SY5Y and HDN. SH-SY5Y human neuroblastoma cells were treated with RA for 48 h. Statistics are given as mean ± SEM. Differences were assessed by one-way ANOVA. ***P* < 0.01, ****P* < 0.001, *****P* < 0.0001. *n* = 350 for MDNCs, *n* = 234 for SH-SY5Y and *n* = 83 for human neurons.

TABLE S1 Single cell mRNA sequencing of 17 cells exposed to our transdifferentiation protocol.
