# Supplementary figures and images for: Transdifferentiation of Human Circulating Monocytes Into Neuronal-Like Cells in 20 Days and Without Reprograming
Source: Front Mol Neurosci. 2018 Sep 19;11:323. doi: 10.3389/fnmol.2018.00323 (PMC6156467; doi:10.3389/fnmol.2018.00323)

# Supplementary Figure 1

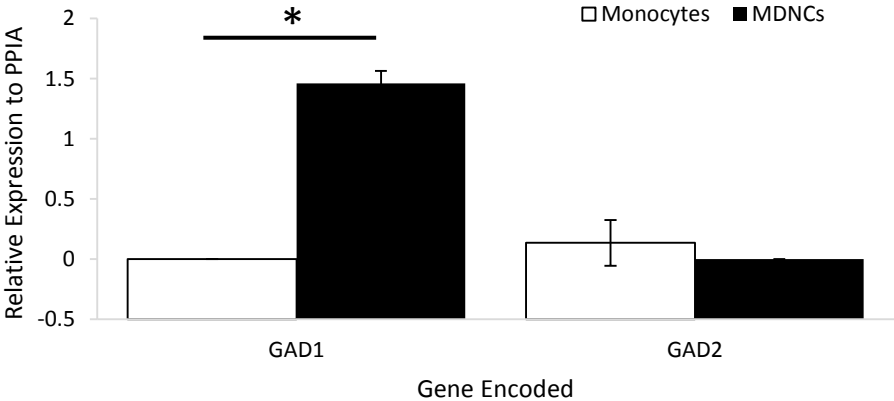

## Supplementary Figure 2

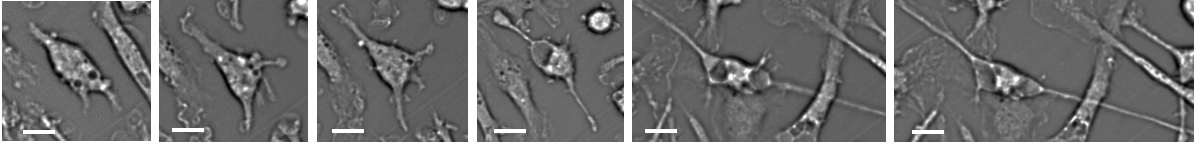

## Supplementary Figure 3

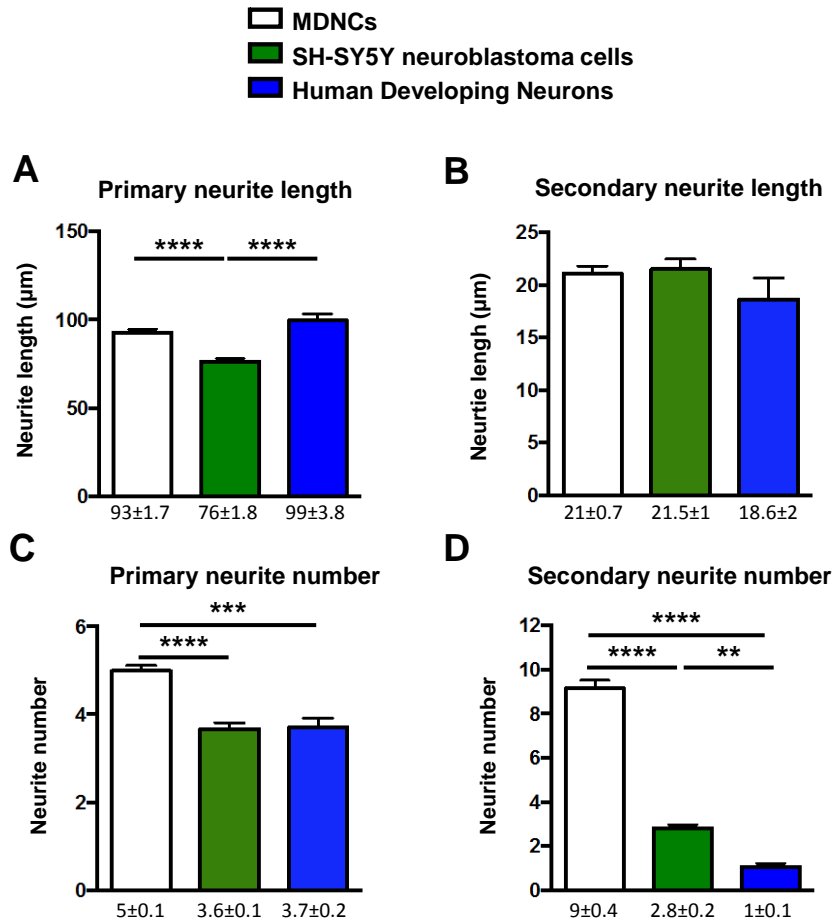

Supplement: FIGURE S1 — Glutamic Acid Decarboxylase (GAD) gene expression in human circulating monocytes transdifferentiated into neural-like cells. Bar graphs depicting expression of Glutamic Acid Decarboxylase 1 and 2 (GAD1 and GAD2) relative to Cyclophilin A (PPIA), which was used as a reference gene. GAD1 encodes for GAD67 and GAD2 encodes for GAD65. Both proteins are considered markers for GABAergic neurons. GAD1 was expressed in monocyte-derived-neuronal-like cells (MDNCs) but not evident in undifferentiated monocytes (*P < 0.029). GAD2 is not expressed in either MDNCs or monocytes. These bars represent four experiments done by triplicate with cells from three different healthy subjects. [file Data_Sheet_1.pdf]
