# Supplementary material for: Transdifferentiation of Human Circulating Monocytes Into Neuronal-Like Cells in 20 Days and Without Reprograming
Source: Front Mol Neurosci. 2018 Sep 19;11:323. doi: 10.3389/fnmol.2018.00323 (PMC6156467; doi:10.3389/fnmol.2018.00323)
Supplement: TABLE S1 — Single cell mRNA sequencing of 17 cells exposed to our transdifferentiation protocol. [file Table_1.pdf]

**Supplementary Table 1.** Single cell mRNA sequencing of 17 cells exposed to our transdifferentiation protocol.

**Sequenced cell***

| Explanation | Marker type | Coding number | Gene symbol | a | b | c | d | e | f | g | h | i | j | k | l | m | n | o | p | q | SH-SY5Y** |
|---|---|---|---|---|---|---|---|---|---|---|---|---|---|---|---|---|---|---|---|---|---|
| NeuN transcription factor | mature neurons | ENSG00000167281 | *RBFOX3* | 0.26 | 0.27 | 1.19 | 1.24 | 0.24 | 0.16 | 0.56 | 0.85 | 1.86 | 0.44 | 0.36 | 0.55 | 0.29 | 0.86 | 0.88 | 3.50 | 0.65 | 0.4 |
| vGLUT1 | glutamatergic neurons | ENSG00000104888 | *SLC17A7* | | | | | | | | | | | | | | | | | | 0.4 |
| vGLUT2 | glutamatergic neurons | ENSG00000091664 | *SLC17A6* | | | | | | | | | | | | | | | | | | 0 |
| NMDAR1 | glutamatergic neurons | ENSG00000176884 | *GRIN1* | | 0.56 | | | | | | | | | | | | | | | | 4.2 |
| NMDAR2B | glutamatergic neurons | ENSG00000273079 | *GRIN2B* | | | | | | | | | | | | | | | | | | 0 |
| Glutaminase | glutamatergic neurons | ENSG00000115419 | *GLS* | 1.39 | 0.29 | 1.33 | | 1.80 | 2.12 | 1.25 | 65.8 | 22.5 | 1.16 | 87.90 | | 0.98 | 22.1 | 13.9 | | 6.42 | 49.6 |
| GABA transporter 1 | gabaergic neurons | ENSG00000157103 | *SLC6A1* | | | | | | | | | | | | | | | | | | 0 |
| GABA type B receptor, subunit 1 | gabaergic neurons | ENSG00000204681 | *GABBR1* | | | | | | | | | | | | | | | | | | 1.1 |
| GABA type B receptor, subunit 2 | gabaergic neurons | ENSG00000136928 | *GABBR2* | | | | | | | | | | | | | | | | | | 0 |
| Glutamate decarboxylase 1 (67KDa) | gabaergic neurons | ENSG00000128683 | *GAD1* | | | 0.90 | | | | | | | | | | | | | | | 2.1 |
| Glutamate decarboxylase 2 (65KDa) | gabaergic neurons | ENSG00000136750 | *GAD2* | | | | | | | | | | | | | | | | | | 0 |
| Tyrosine hydroxylase | dopaminergic neurons | ENSG00000180176 | *TH* | | | | | | | | | | | | | | | | | | 0 |
| DAT, Dopamine Transporter | dopaminergic neurons | ENSG00000142319 | *SLC6A3* | | | | | | | | | | | | | | | | | | 0 |
| A transcriptional activator that regulates differentiation of dopaminergic neurons | dopaminergic neurons | ENSG00000125798 | *FOXA2* | | | | | | | | | | | | | | | | | | 0 |
| Girk2, a G-protein present in certain dopaminergic neurons | dopaminergic neurons | ENSG00000157542 | *KCNJ6* | 0.67 | 0.13 | 0.48 | 0.80 | 0.31 | 0.33 | 0.24 | 0.95 | 0.24 | 0.28 | | 0.14 | 0.18 | 0.50 | 0.14 | 0.33 | 0.46 | 0 |
| Nurr1, a transcription factor that induces TH expression and subsequently dopaminergic neuron differentiation | dopaminergic neurons | ENSG00000153234 | *NR4A2* | | | | | | 0.18 | | | | | 7.38 | | | 28.1 | | | | 0.1 |
| A transcription factor involved in a number of processes during dopaminergic neuron development | dopaminergic neurons | ENSG00000136944 | *LMX1B* | 0.19 | 0.40 | 0.43 | 1.00 | | | 0.30 | 0.18 | 0.42 | 0.26 | | 0.25 | 0.95 | 0.17 | 0.82 | | 0.14 | 1.6 |
| Tryptophan hydroxylase, an enzyme involved in serotonin synthesis | serotonergic neurons | ENSG00000129167 | *TPH1* | 0.63 | 0.20 | 59.30 | 0.79 | 0.45 | | 0.35 | 0.50 | 0.14 | 0.49 | 0.16 | 0.54 | 0.14 | 0.38 | 0.21 | | 0.12 | 0.3 |
| Tryptophan hydroxylase, an enzyme involved in serotonin synthesis | serotonergic neurons | ENSG00000139287 | *TPH2* | 0.39 | 0.12 | 0.86 | | 0.22 | | | | | 0.79 | | | | 0.17 | 0.20 | | 0.77 | 0 |
| Serotonin transporter | serotonergic neurons | ENSG00000108576 | *SLC6A4* | | | 0.19 | 0.49 | 0.95 | | 0.15 | | 0.38 | 0.14 | | | | 0.12 | | | 0.28 | 0 |
| PET1, transcription repressor implicated in the differentiation of serotonergic neurons | serotonergic neurons | ENSG00000163497 | *FEV* | | | | | | | | | | | | | | | | | | 34.6 |
| Cholin acetyltransferase | cholinergic neurons | ENSG00000070748 | *CHAT* | | | | | | | | | | | | | | | | | | 0 |
| VAChT | cholinergic neurons | ENSG00000187714 | *SLC18A3* | | | | | | | | | | | | | | | | | | 7.6 |
| acetylcholinesterase | cholinergic neurons | ENSG00000087085 | *ACHE* | | | 0.24 | | | | | | | | | | | | | | | 2.1 |
| Insulin gene enhancer protein | motor neurons | ENSG00000016082 | *ISL1* | | | | | | | | | | | | | | | | | | 307.2 |
| Motor neurons and pancreas homeobox 1 | motor neurons | ENSG00000130675 | *MNX1* | | | | | | | | | | | | | | | | | | 0 |

*Empty boxes correspond to a level of expression of zero.
**Human neuroblastoma cells for comparison (data taken from the Human Protein Atlas database).
